# Aging of *Xenopus tropicalis* Eggs Leads to Deadenylation of a Specific Set of Maternal mRNAs and Loss of Developmental Potential

**DOI:** 10.1371/journal.pone.0013532

**Published:** 2010-10-22

**Authors:** Anna Kosubek, Ludger Klein-Hitpass, Katrin Rademacher, Bernhard Horsthemke, Gerhart U. Ryffel

**Affiliations:** 1 Institut für Zellbiologie (Tumorforschung), Universitätsklinikum Essen, Universität Duisburg-Essen, Essen, Germany; 2 Institut für Humangenetik, Universitätsklinikum Essen, Universität Duisburg-Essen, Essen, Germany; Radboud University Nijmegen, Netherlands

## Abstract

As first shown more than 100 years ago, fertilization of an aged (overripe) egg increases the rate of malformations and embryonic loss in several vertebrates, including possibly humans as well. Since the molecular events in aging eggs may be similar in these species, we established in the frog *Xenopus tropicalis* a defined protocol for delayed fertilization of eggs. A three-hour delayed fertilization led to a dramatic increase in malformation and mortality. Gene expression profiling revealed that 14% of the polyadenylated maternal transcripts were downregulated upon aging. These transcripts were not degraded, but rather deadenylated as shown for specific maternal mRNAs. The affected transcripts are characterized by a relatively short 3′UTR and a paucity of cytoplasmic polyadenylation elements (CPE) and polyadenylation signals (PAS). Furthermore, maternal mRNAs known to be deadenylated during egg maturation as well as after fertilization were preferentially deadenylated in aged eggs. Taken together our analysis of aging eggs reveals that unfertilized eggs are in a dynamic state that was previously not realized. On the one hand deadenylation of transcripts that are typically deadenylated during egg maturation continues and this implies overripeness of the aged egg in the truest sense of the word. On the other hand transcripts that normally are deadenylated after fertilization loose their poly(A) in the aged egg and this implies that the egg awaiting fertilization starts processes that are normally only observed after fertilization. Based on our novel finding we postulate that the imbalance of the polyadenylated maternal transcripts upon egg aging contributes to the loss of developmental potential. Based on this hypothesis the developmental consequences of downregulation of specific transcripts can be analyzed in future.

## Introduction

Egg quality is acquired during oocyte growth and maturation and can be defined as the ability of the egg to be fertilized and subsequently develop into a normal embryo. Ovulated oocytes, i.e. eggs, are arrested at metaphase II and are normally fertilized within a few hours. Although the terms “oocyte” and “egg” are used quite differently in various species, we use for clarity the term “egg” for the ovulated female gamete arrested in metaphase II. More than 100 years ago Pflüger reported that delayed fertilization of frog eggs (*Rana temporaria)* resulted in a high incidence of abnormal development [1]. This observation was not only confirmed in other frogs [2], but also in rainbow trout [3], mouse [4], rat [5] and guinea pig [6]. In agreement, in humans the risk of early pregnancy loss has been reported to be increased with the probability that the egg has aged before being fertilized [7]. Today the issue of delayed fertilization and a potential increase in malformations is most important in humans, since up to 4% of all babies in Europe are born following Assisted Reproduction Technologies (ART) [8]. Although ART has been progressively improved over the last 30 years, a 30–40% increased risk of birth defects for ART children has been reported [9]. Factors suggested to be responsible for the increased risk are the underlying causes for infertility in the couples receiving ART, but also factors associated with the techniques *per se* that influence egg quality [9]. Indeed, Jongbloet was the first to suggest that overripeness may contribute to the adverse outcome of ART [10]. Thus, eggs in the oviduct or in culture are likely to undergo a time dependent quality loss, a process also called postovulatory oocyte aging [11] and it is most relevant to understand the molecular events involved in the declined potential in aged eggs.

Although many changes in morphology and cell biology during egg aging [11] and specific epigenetic alterations have been described [12], [13], no global analysis on the level of gene expression has been performed. Since in all vertebrates transcription starts only after egg cleavage, aging induced alterations may involve changes in maternal mRNAs that have been deposited during oocyte maturation. In recent years research has shown that in oogenesis and early embryogenesis translational control plays a major role including temporal regulation of cytoplasmic polyadenylation and deadenylation [14], [15] mediated via multiple cis-acting elements in the 3′UTR of mRNAs [16]. Further, a study in rainbow trout has indicated that egg aging is associated with variations in the relative abundance of several maternal mRNAs. [17] Therefore, we speculate that postovulatory aging disturbs the pool of maternal transcripts.

To address this experimentally we have chosen the frog *Xenopus*, because its development constitutes an excellent model for embryogenesis of vertebrates [18]. Hundreds of eggs are available, early development reaching complete organogenesis is attained within four days and external development in water allows visual inspection and easy manipulation. In addition, most control mechanisms that operate in mammalian oogenesis have been first and most intensively studied in *Xenopus laevis*. As genomic analysis of *Xenopus laevis* is complicated due to its pseudo-tetraploid genome, we used for our investigation *Xenopus tropicalis* which shares the positive features of its close relative, but has the advantage of being a truly diploid species and thus genome wide analysis is considerably facilitated [19] and a draft genome assembly has been reported most recently [20]. Furthermore, gene expression profiling in *Xenopus tropicalis* has defined seven categories of maternal mRNAs with distinct adenylation behavior upon egg maturation and fertilization [15]. Because of this basic knowledge of differential polyadenylation and deadenylation of maternal mRNAs prior to the onset of zygotic transcription, it is sensible to study postovulatory aging in this species.

## Results

### Loss of egg potential upon postovulatory aging

**Figure 1:**
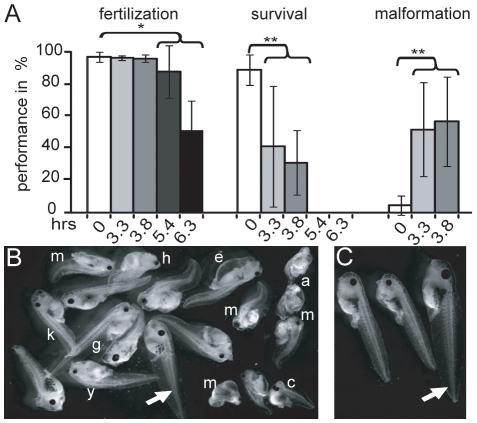
Postovulatory aging effect on egg quality. (A) Batches of 100–300 eggs were fertilized immediately (white bars) or with the delay as indicated (light to dark grey bars). The fertilization rate was scored at the 4-cell stage 3 [21], whereas the survival and malformation rates were determined at the free swimming larval stage 40 (20). At 0, 3.3, 3.8, 5.4 and 6.3 hours 7, 3, 3, 2 and 3 females were used, respectively. Error bars represent standard deviation and the values of the groups indicated by the bracket were compared to the immediately fertilized sample using student t-test (* and **denote p-values of <0.05 and 0.01, respectively). (B) Malformation observed at free swimming larvae: edema (e), abnormal gut formation (g), truncated head (h), small eye (y), kinky tails (k), acephalus (a), cyclop (c) and multiple deformations (m) recorded from different experiments. Normal larvae indicated by arrow. (C) Additionally some larvae without malformations were undersized and retarded in development. Arrow indicates normal larvae.

To study whether postovulatory aging affects the developmental potential of the *Xenopus tropicalis* egg we fertilized at different time intervals egg aliquots made from batches of eggs collected from hormonally primed females. Up to 3.8 hours the fertilization rate stayed constant and then dropped significantly (Fig. 1A). In contrast, the survival recorded at the swimming larval stage 40 [21] dropped by about two-fold at a 3.3-hour delayed fertilization and no larvae survived at 5.4 hours. This drop in survival was accompanied by a dramatic increase in malformation. The abnormal embryonic development included a large variation of malformations like edema, acephaly, compressed and distorted spinal cord and cyclops (Fig. 1B). In addition, many larvae that showed no malformations upon egg aging were undersized and retarded in development (Fig. 1C). For subsequent experiments we chose a 3-hour delay in fertilization, since under these conditions malformation increases significantly, but fertilization rate remains high enough to assure evaluation of the developmental performance.

### Changes in the maternal polyadenylated mRNA population in aged eggs

Since no transcriptional activity occurs prior to fertilization [22], we postulate that the decreased potential of aged eggs reflects alterations of some maternal components. To address this issue we focused on the maternal polyadenylated mRNA population using Affymetrix *Xenopus tropicalis* Genome Array containing more than 58,000 probesets representing over 51,000 transcripts. We compared fresh *versus* 3-hour aged unfertilized eggs from a batch of eggs, where the larvae derived from aged eggs exhibited a 2.5-fold increase of malformation and a 4.2-fold increase including underdevelopment compared to immediately fertilized eggs (Fig. S1A). To account for biological variation in a spawn we analyzed two pools of ten fresh eggs versus two pools of ten 3-hour aged eggs and the cross comparison of the microarray data show highly correlating replicates (Fig. S1B). For further analysis only probesets that showed a consistent change in all four comparisons were taken into account. Using these criteria we identified 1785 signal decreased probesets that include 897 and 177 probesets decreased by more than two- and four-fold, respectively. Only 174 probesets showed an increased signal, including nine with a two-fold change. Overall about 15% of the probesets that detect transcripts in fresh eggs exhibited an alteration upon aging.

**Figure 2:**
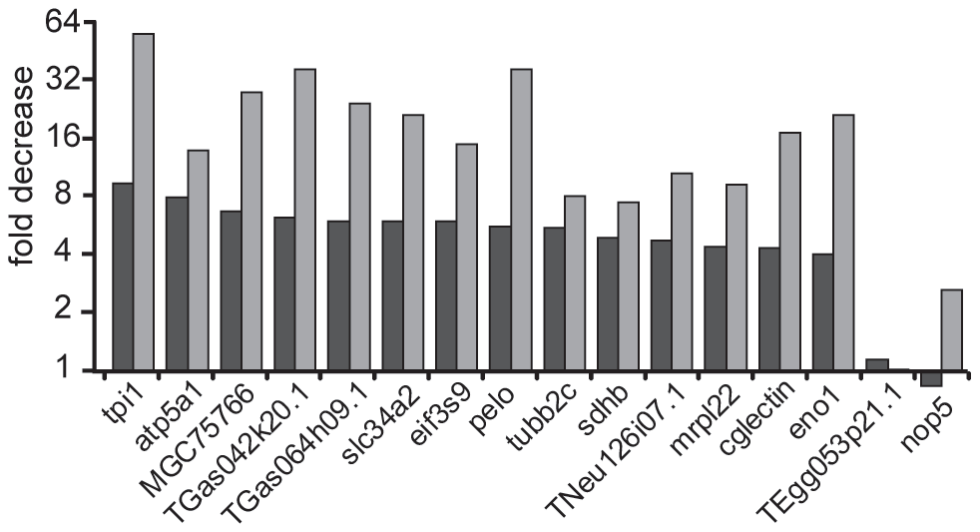
Quantification of specific polyadenylated transcripts upon egg aging. The fold decrease in the relative amount of polyadenylated transcript in eggs aged for 3 hours compared to immediately fertilized eggs is indicated as obtained from microarrays (black bars) and oligo(dT) primed qRT-PCR (grey bars). The transcripts are named according to the corresponding gene symbols listed in Table S1 and were selected based on their >4-fold decrease in the microarray upon egg aging. TEgg053p21.1 and nop5 are classified by microarray analysis as transcripts with no change upon egg aging and are used as controls.

To confirm the alterations observed by microarray analysis we performed qRT-PCR using oligo(dT) primed cDNA synthesis on the same RNA preparation as used for microarrays. As in both methods we used oligo(dT)_20_ primers for the reverse transcription, only changes in the poly(A) length that shorten the poly(A) to less than 20 As were assessable. We selected fourteen transcripts that were at least four-fold decreased, full-length and annotated (Table S1). As assayed by qRT-PCR all these transcripts were decreased and the fold decrease was even stronger than observed by microarrays (Fig. 2). In conclusion, the data derived from both assays are in good agreement.

### The drop in egg potential correlates with downregulation of polyadenylated transcripts

**Figure 3:**
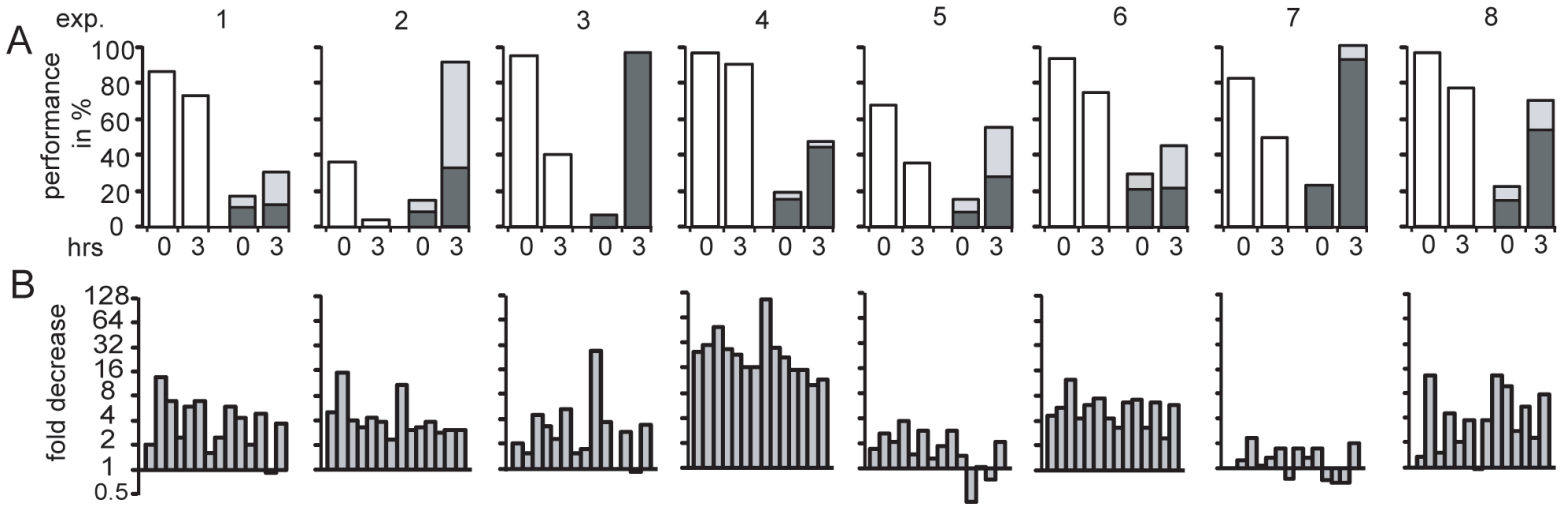
Drop in egg potential and decrease in specific polyadenylated transcripts upon egg aging in batches of eggs obtained from different frogs. Eggs were fertilized immediately or with a 3-hour delay. (A) The developmental competence refers to the survival rate (white boxes) and the percentage of defective development (grey boxes) observed at the free swimming larval stage 40 (20). The defective development includes malformation (dark grey) as well as undersized and retarded development (light grey) as exemplified in Fig. 1B and C. Experiment (exp.) 6 and the experiment in Fig. 2 were made ten months apart using the same female. (B) From each experiment the aging induced decrease determined by oligo(dT) qRT-PCR of the fourteen polyadenylated transcripts is given in the same order as analyzed in Fig. 2.

To evaluate the variability of egg aging in individual females we used eggs from eight females. The comparison of these eight females reveals the influence of egg quality on survival and development (Fig. 3A) indicating that many factors play a role. However, in all experiments we observed a drop of larvae survival and an increase in the proportion of defective larvae upon aging. Quantification by RT-PCR of the fourteen transcripts revealed in most cases a decrease in these transcripts, although there was no quantitative correlation between the loss of developmental potential and the decrease of the transcripts (Fig. 3B). These data show that the reduced developmental potential of aged eggs is highly reproducible and suggest the downregulation of polyadenylated transcripts as one but possibly not the only causative factor.

In the experiments described so far we used modified Barth solution with 0.5% bovine serum albumin (BSA) for storing the eggs. In this high salt medium the jelly coat does not swell and thus the eggs can still be fertilized several hours later [18]. To exclude that the observed signal-reduction results from incubation in salt solution, but not from egg aging *per se*, we stored the eggs in a wet chamber that also prevents the formation of the jelly coat. Also under these conditions we detected a decrease in survival and an increase in malformations that was correlated with a drop in the abundance of the fourteen transcripts (Fig. S2).

**Figure 4:**
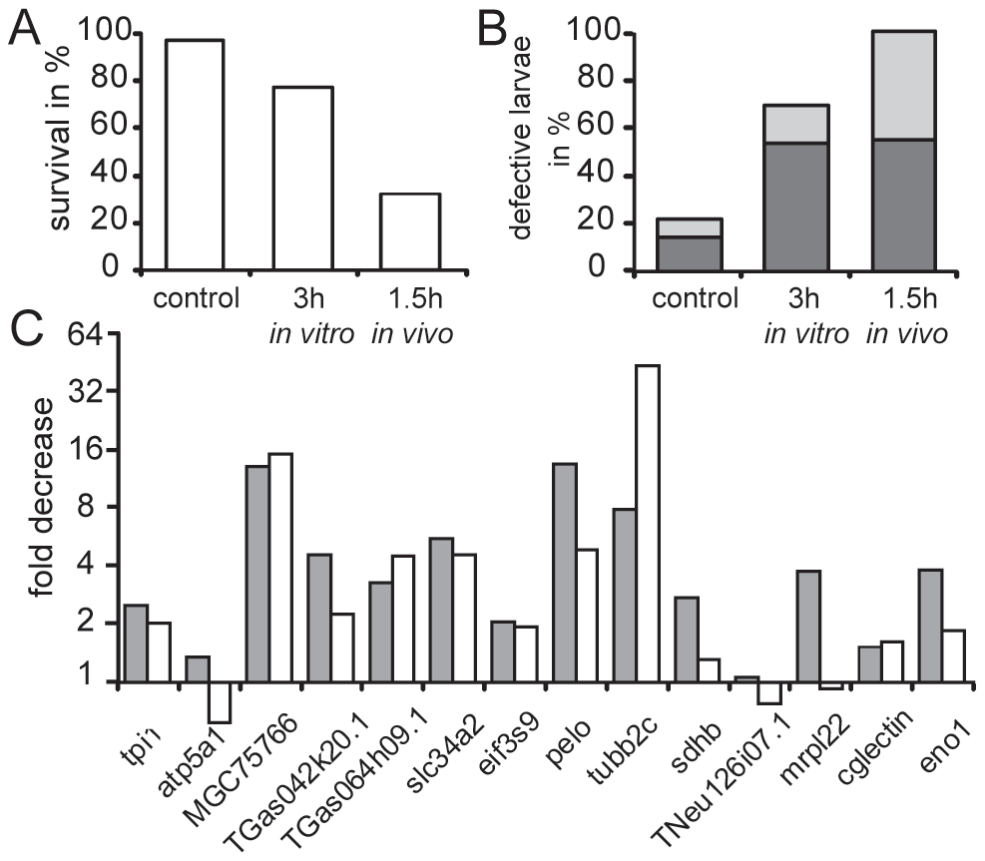
Comparison of *in vitro* and *in vivo* egg aging. (A–C) Eggs were fertilized immediately, with a 3-hour delay (*in vitro*) or from a batch obtained 1.5 hours later (*in vivo*) from the same female shown also in Fig. 3 as exp. 8. The survival rate (A) and the percentage of defective development (B) observed at the free swimming larval stage 40 [21] is given. The defective development includes malformation (dark grey) as well as undersized and retarded development (light grey). (C) From each experiment the aging induced decrease of the fourteen polyadenylated transcripts analyzed in Fig. 2 is given (gray boxes: *in vitro* aging; white boxes *in vivo* aging).

To analyze whether the monitored alterations of *in vitro* aged eggs may represent changes that occur also *in vivo,* we compared eggs collected directly after ovulation to eggs squeezed from the same female 1.5 hours later. We observed that immediately fertilized eggs from the second spawn showed also a high loss in development potential like eggs from the first spawn aged *in vitro* for 3 hours (Fig. 4A–B). As under both conditions a similar decrease of the transcripts was observed (Fig. 4C), it seems possible that eggs harvested later have a lower quality reflecting *in vivo* aging. This is a general finding when collecting eggs from hormonally primed Xenopus [18]. However, a direct correlation between the fold decrease for the transcripts and the degree of quality loss is not given implying additional factors determining developmental potential.

### Deadenylation explains the decreased transcript signals

**Figure 5:**
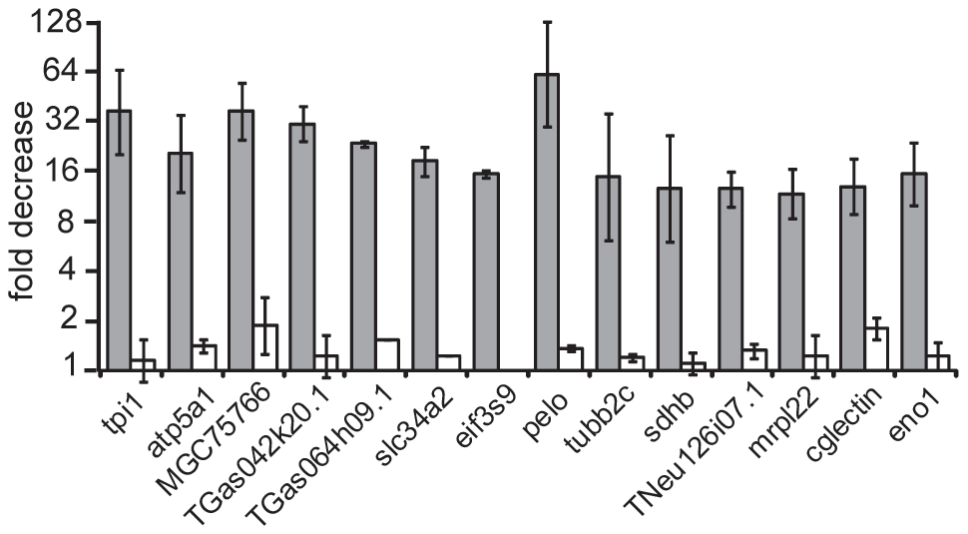
Deadenylation upon egg aging. The fold decrease in relative abundance of the fourteen polyadenylated transcript in aged eggs measured by qRT-PCR using oligo(dT) (grey bars) or random primed cDNA (white bars) is given for two females, the same as used for Fig. 2 and exp.4 in Fig. 3. Error bars represent standard deviation.

As our microarrays and the qRT-PCR using oligo(dT) quantify polyadenylated transcripts exclusively, the decreased abundance of polyadenylated mRNA could reflect either deadenylation or degradation of maternal mRNA. Therefore, we compared the quantification of the fourteen transcripts by qRT-PCR using either oligo(dT) or random primers. As with random primed cDNA no significant changes were detected (Fig. 5), we deduce that the reduced transcript signals reflect deadenylation and not degradation of maternal transcripts.

**Figure 6:**
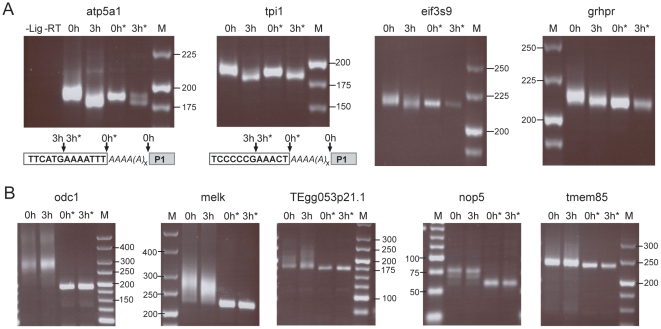
Poly(A) tail reduction of specific maternal mRNA in aged eggs. Poly(A) tail behavior of the indicated transcripts decreased (A) and not changed (B) upon egg aging are shown. Total mRNA from fresh (0 h) and aged (3 h) eggs was assayed by the RNA ligation-mediated poly(A) test (RL-PAT). * indicates RNaseH/oligo(dT)_20_ digestion prior to ligation. Control lanes: –Lig, Ligation reaction performed without RNA; -RT, ligated RNA was not reverse transcribed prior to PCR. M, DNA size marker are given in base pairs. Direct sequencing of atp5a1 and tpi1 (A lower panel) reveals the actual transcript 3′ending (indicated by arrows), which is in fresh eggs at the end of the poly(A) tail (italic As), but in aged eggs several nucleotides upstream of the former end of the RNA body (clear box). P1 is the ligated primer.

To show directly that the decrease in transcripts relies on a shortening of the poly(A) tail we used the RNA ligation-mediated poly(A) test (RL-PAT) [23] of decreased (Fig. 6A) and not-changed (Fig. 6B) transcripts. Aged eggs revealed a poly(A) tail shortening in all four decreased transcripts. Notably transcripts of aged eggs were shorter than transcripts of fresh eggs deadenylated by RNaseH/oligo(dT)_20_ treatment (Fig. 6A). Direct sequencing laid open a complete deadenylation plus a loss of seven and five nucleotides of the RNA body in atp5a1 and tpi1, respectively. In contrast, transcripts being not changed upon aging showed no transcript shortening, independent of the poly(A) length in fresh eggs (Fig. 6B).

### Transcript signal reduction correlates with a short 3′UTR and a paucity of CPE as well as of PAS

**Figure 7:**
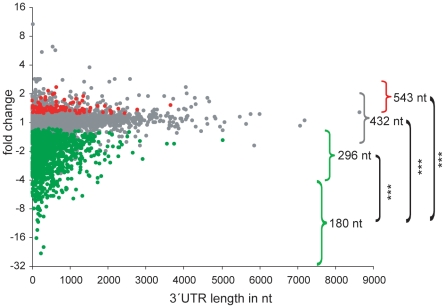
Transcript signal reduction correlates with a shorter 3′UTR length. The average signal fold change of probesets, which were scored as consistently increased, not changed and decreased in all 4 cross-comparisons, were plotted against the length of the 3′UTR. Probesets receiving decreased (N = 1134), not changed (N = 4050) and increased change (N = 107) calls in the Affymetrix comparison analysis (MAS 5.0 statistical algorithm) are given in green, grey and red, respectively. Transcripts classified according to signal alterations upon aging, i.e. >4-fold decrease, <4-fold decrease, not changed and increased, are marked with brackets and the median length of the 3′UTR of each class is given. Highly significant p-values between >4-fold decreased transcripts and all other classes derived by Mann-Whitney test are indicated (*** p-value<0.001).

To identify common features of the signal-reduced transcripts we correlated the length of the 3′UTR sequences with the degree of downregulation for all probes with a full-length annotation given in the UCSC browser. Interestingly, we observed an inverse correlation between the magnitude of signal reduction and the 3′UTR length (Fig. 7). In fact, more than four-fold signal-decreased transcripts exhibited a median 3′UTR length of 180 nt that was significantly shorter than the median length of 296 nt, 432 nt or of 543 nt of transcripts less than four-fold decreased, with no change or increased, respectively (Fig. 7).

The cytoplasmic polyadenylation element (CPE) and the poly(A) signal (PAS) are both involved in polyadenylation behaviour of mRNA [24]. Since these elements are usually close to the 3′ terminus, we scored for the presence of PAS and CPE in the 3′ terminal 100 and 120 nucleotides, respectively, of each full-length transcript and correlated this frequency with the extent of signal reduction. To allow a meaningful interpretation we classified the decreased transcripts into three categories with distinct extent of signal reduction and used as fourth category all transcripts with an increased signal.

**Figure 8:**
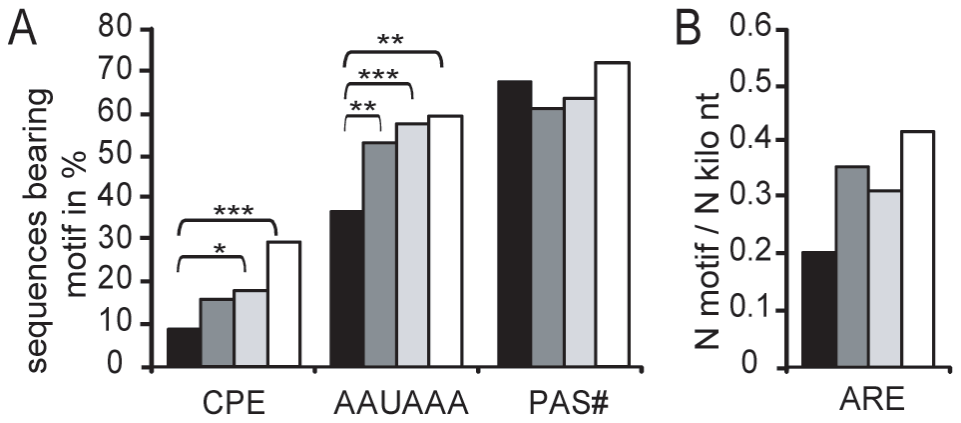
Abundance of CPE, PAS and ARE in the 3′UTR of transcripts deadenylated upon egg aging. Transcripts were classified according to their signal alteration magnitude: >4-fold decreased (black, N = 98), 4>x>2-fold decreased (dark grey, N = 403), <2-fold decreased (light grey, N = 496) and increased (white, N = 96). (A) Percentage of mRNA sequences bearing at least one canonical PAS (AAUAAA), one non-canonical PAS (PAS#) or one CPE in the 3′terminal 100nt for each transcript category are given. *, ** and *** denote p-values of <0.05, p<0.01 and p<0.001 using Fisher's exact test, respectively. (B) Abundance of ARE in the entire 3′UTR was normalized against the total 3′UTR length of the corresponding transcript class and analyzed using Mann-Whitney test.

We considered six alternative sequences that have been defined as CPE (Table S2) and found a decreased frequency in the transcripts with a more than four-fold reduced signal compared to other categories (Fig. 8A). A similar paucity was also found for the canonical PAS AAUAAA represented in about 60% of the mRNA [25]. In contrast, the less frequent non-canonical PAS (Table S2) were distributed equally in the 3′UTR sequences of all categories (Fig. 8A).

Since AU rich element (ARE) known as destabilizing element is not reported to be at a specific region of the 3′UTR, six as ARE referred motifs (Table S2) were scored in the entire 3′UTR. After normalization of their occurrence to the total 3′UTR length of the corresponding category we found no significant enrichment in any category (Fig. 8B).

### Transcripts deadenylated at oocyte maturation and after fertilization are enriched for transcripts deadenylated upon egg aging

**Figure 9:**
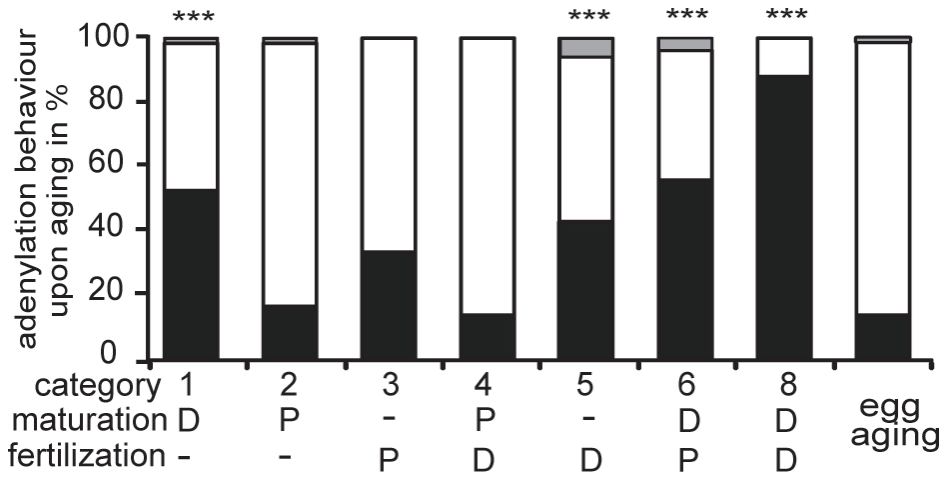
Distribution of maternal transcripts deadenylated upon egg aging to the RNA categories with distinct adenylation behavior at egg maturation and upon fertilization. The transcripts of each category defined according to their deadenylation and/or polyadenylation were classified in percentage whether they are decreased (black), not changed (white) or increased (grey) upon egg aging. Deadenylation (D) and polyadenylation (P) at egg maturation and upon fertilization for each category is given. Category 7 is not shown as this type of behavior was not observed [15]. For comparison the percent distribution of decreased (black), not changed (white) and increased (grey) transcripts in aged eggs of all transcripts measurable by Affymetrix microarrays is given. Statistically significant differences in the distribution of the categories compared to all measured transcripts are indicated by three asterixs (p<0.0001 by chi square statistics).

A comparison of the transcripts deadenylated in aged eggs with the seven categories defined by their differential adenylation behaviour [15] revealed a significantly uneven distribution of transcripts deadenylated upon egg aging (Fig. 9). Indeed, categories 1, 6 and 8 defined by their deadenylation during egg maturation include a significant high proportion of probesets of transcripts deadenylated upon egg aging. A similar significant overrepresentation was also seen in category 5 characterized by transcripts deadenylated after fertilization, whereas all other categories (2, 3 and 4) showed no enrichment. Transcripts deadenylated at egg maturation and after fertilization that are also decreased upon egg aging, are derived from genes with many different functions (Table S3).

## Discussion

By using *Xenopus tropicalis* we have established an excellent model for studying egg aging and have revealed changes in maternal transcripts that can not easily be explored in other species. So far only preovulatory aging has been analyzed in *Xenopus laevis* that leads to high mortality and various malformations [26] comparable to what we observed here in postovulatory aging.

Investigating the effect of delayed fertilization in *Xenopus tropicalis* eggs is very attractive due to its simplicity, as it involves egg storing in a salt solution or in a wet chamber. In contrast to preovulatory aging studies, inter-female variations and influences by female age and breeding conditions including feeding, water temperature and composition, can be excluded for a single postovulatory aging experiment, as batches of up to a thousand eggs are harvested simultaneously at the same time from a single female and can be distributed into aliquots for defined *in vitro* aging. Thus, expression profiling of eggs from the same spawn reveals a highly reproducible pattern (Fig. S1B), while substantial inter-female variation occurs in the biological response as well as in the downregulation of specific transcripts comparing eight different females (Fig. 3).

Although aging of *Xenopus tropicalis* eggs for at least up to 4 hours neither changes egg pigmentation indicating artificial activation nor alters fertilization rate (Fig. 1), delayed egg fertilization leads to malformations and a progressive increase in mortality. The increased death rate reflects serious developmental defects such as incomplete gastrulation and neurulation that are frequently incompatible with further development and thus lead to early death. We observed a similar drop in egg potential upon postovulatory aging in the related species *Xenopus laevis* (Fig. S3). In these experiments we also excluded that sperm aging has an effect, as the performance with fresh and 5-hour aged sperms was indistinguishable. A comparison of *in vivo versus in vitro* egg aging implies a most similar effect (Fig. 4). However, we can not exclude that a batch of eggs harvested later may be distinct, although it is from the same female, as not all the eggs mature simultaneously and may be derived from oocytes of distinct quality. Based on this uncertainty we used *in vitro* aged eggs for our experiments. Our data establish that *Xenopus* has the main feature of impaired development upon postovulatory aging well documented from fish to mammals [1]–[6] including human [7], making *Xenopus* well suited to investigate mechanisms involved in egg aging. 3-hour aging in *Xenopus tropicalis* seems a short time period. However, as the development is very fast, it reflects the time needed to attain the early morula stage [27], which is reached in mouse within three days [28].

We show for the first time using microarray and qRT-PCR that the decreased egg potential upon postovulatory aging is reflected in a decrease of 14% of the maternal polyadenylated mRNA. This proves that changes occur in the molecular outfit of the *Xenopus* egg within a 3-hour aging period. Because significant alterations are detected only in qRT-PCR using oligo(dT), the apparent signal reduction implies RNA deadenylation in the absence of degradation (Fig. 5) and this was directly proven by measurements of the poly(A) tails lengths (Fig. 6). The analysis of four random transcripts of the downregulated fraction revealed a short poly(A) that was completely removed upon egg aging (Fig. 6A). Our finding that this shortening even included a few nucleotides of the RNA body is consistent with the reported activity of deadenylases [29], [30]. In contrast, transcripts not changed upon egg aging (Fig. 6B) contained either long poly(A) tails (odc1 and melk) or short poly(A) tails (TEgg053p21.1, nop5 and tmem85). We deduce that during egg aging selective deadenylation of specific maternal mRNAs occurs and that a shortening of the poly(A) to less than 20 nucleotides impairs oligo(dT) mediated reverse transcription needed to detect the corresponding transcript. The loss of poly(A) without a corresponding disappearance of the mRNA may be not too surprising, since unlike in most cell type deadenylation of mRNA in eggs does not trigger transcript degradation. Typically, in eggs deadenylated mRNAs remain stable but dormant until they get polyadenylated again to be translated [16].

In the rainbow trout changes in the abundance of eight polyadenylated transcripts upon postovulatory aging have been reported using random primers on isolated polyadenylated RNA suggesting a change in the concentration of these mRNAs [17]. However, since the non-polyadenylated fraction was not analyzed, it could well be that these RNAs were deadenylated and thus shifted into the non-polyadenylated fraction. In several studies gene expression profiling was done on oocytes matured under different conditions [31]–[33]. As in these studies preovulatory differences are inherent, a direct comparison with our data is not appropriate, but we note that potential changes in the polyadenylation state have been ignored in these investigations.

As in *Xenopus* but also in mouse eggs deadenylated mRNAs are poorly translated [16], we suppose that the deadenylation of a specific set of maternal RNAs in aged eggs leads to corresponding reduced synthesis of specific proteins. To get some insight into the possible functions impaired by postovulatory egg aging we used the Gene Ontology Enrichment Analysis Software Toolkit [34]. However, this approach is not optimal for *Xenopus tropicalis*, as only a limited number of reliable annotations are available. This drawback impedes a detailed comparison to other species such as mouse and human with reliably annotated genes. With this limitation in mind we found among others the strongest overrepresentation in the categories of “translation” and “energy metabolism”. In fact, similar gene ontology enrichment has been reported for maternal RNA obtained from mouse, bovine and *Xenopus laevis* [35] as well as from human oocytes [33].

There are several distinct pathways of deadenylation in oocytes and early embryos that have been best characterized in *Xenopus* and are considered to be most relevant in mammals as well [36]. The first pathway is the “default” deadenylation in maturing *Xenopus* oocytes [37] that is relatively slow. Since the polyadenylated transcripts downregulated upon postovulatory aging contain CPE at a decreased level, a feature unique to this pathway, we speculate that this process is responsible for the decrease in maternal transcripts observed in our aging experiments. In contrast, we rather exclude the second mechanism that represent a targeted deadenylation involving ARE [38], since we did not find an overrepresentation in ARE in the maternal transcripts deadenylated upon aging. The third pathway is mediated by the embryonic deadenylation element (EDEN)-specific RNA binding protein [39]. Since we could not find a significant enrichment of the EDEN binding site in the deadenylated transcripts (data not shown), we rather exclude this mechanism, although the low frequency of the EDEN15 motif [40] precludes a thorough statistical analysis. The fourth pathway of deadenylation [41] is most unlikely in the aged eggs, as this process only operates after zygotic gene expression in the blastula stage. Our finding that the most downregulated transcripts contain a short 3′UTR is possibly linked to the fact that most of these transcripts represent housekeeping genes [42]. As translation is regulated via changes in the poly(A) tail length, which is critically controlled by cis-elements in the 3′UTR [24], our finding of a decreased level of the canonical PAS (AAUAAA) and CPE may be most relevant. CPE is the best characterized element critical for cytoplasmic poly(A) tail length regulation conserved from invertebrates to mammals. Its binding protein CPEB can assemble complexes that repress mRNA translation as reported in immature oocytes, or that direct cytoplasmic polyadenylation and activate translation e.g. in maturing oocytes [43], [44]. Maturation specific deadenylation in both amphibian and mouse oocytes is considered as a default pathway for mRNA that lack CPEs [36]. Specifically, in *Xenopus tropicalis* more than 50% of the transcripts that gain a poly(A) tail during oocyte maturation contain CPEs, while transcripts deadenylated during maturation are relatively devoid of CPE [15]. We searched also for PAS elements that are known to be initially needed for cleavage and nuclear adenylation of primary mRNA, but are important for CPE mediated cytoplasmic polyadenylation as well, since the distance of up to 120 nucleotides between the canonical PAS and CPE is essential for this process [44]. Due to this critical neighborhood of these two elements it is not surprising that AAUAAA is underrepresented in the transcripts downregulated by postovulatory aging (Fig. 8A). In contrast, the less frequent non-canonical PAS elements are equally distributed between the various classes of transcripts (Fig. 8A). This correlates with the reports that these variant PAS elements seem to have distinct properties [45].

Since we detected a significant underrepresentation of CPE in the polyadenylated transcripts downregulated upon postovulatory aging, we speculate that postovulatory aging possibly reflects an ongoing of the normal egg maturation process not terminated by fertilization. This assumption is fully supported by our finding that the maternal mRNA categories that are known to be deadenylated upon egg maturation [15] are enriched in transcripts that are decreased due to loss of their poly(A) (Fig. 9). This implies that postovulatory aging of the *Xenopus* egg is best described as overripeness in the truest sense of the word, as the deadenylation of specific transcripts occurring at maturation continues. The fact that a high proportion of transcripts deadenylated after fertilization (category 5) are decreased upon egg aging (Fig. 9) reveals that in the aging egg deadenylation of specific transcripts starts prior to fertilization.

Taken together both these findings show that the unfertilized egg is in a dynamic state with an ongoing and beginning removal of specific mRNA poly(A) tails and thus a delayed fertilized egg contains a distinct set of polyadenylated mRNA. Based on our novel finding we postulate that this imbalance of the polyadenylated maternal transcripts upon egg aging contributes to the loss of developmental potential. Based on this hypothesis the developmental consequences of downregulation of specific transcripts can be analyzed in future. For such experiments *Xenopus* is a most suited model as knock-down of specific transcripts is straightforward by using morpholinos. We also anticipate that comparable changes occur in mammalian systems and these events can now be explored by analyzing the expression of the corresponding genes in aged eggs of mammals.

## Methods

### Gamete collection, fertilization and early development

*Xenopus tropicalis* adults were obtained from the European Xenopus Resource Centre at Portsmouth (UK). We prestimulated the females by injection of 10 units of human chorion gonatropin (HCG, Sigma CG10) and induced ovulation 14 hours later by 100 units HCG. After about 4 hours when the first eggs were spontaneously released we collected by gentle pressure on the lower back and abdomen the eggs into Modified Barth solution (MBS; 88 mM NaCl, 1 mM KCl, 0.7 mM CaCl_2_, 1 mM MgSO_4_, 5 mM HEPES, 2.5 mM NaHCO_3_, 0.7 mM CaCl_2_ [18] containing 0.5% bovine serum albumin (BSA, Sigma A-7888). The eggs of each spawn were mixed, divided into aliquots of about 100–500 eggs and fertilized either immediately or after a three to seven hour storage at 25°C. For fertilization MBS was removed as efficiently as possible and fertilization performed by adding a testis suspension in MBS with 0.5% BSA. For fresh and aged eggs the same sperm suspension prepared in MBS with 0.5% BSA was used and stored at 14°C. Shortly before adding sperm suspension, pools of ten unfertilized eggs were collected from each batch and stored at −80°C for RNA extraction. Fertilization rate was monitored at the four-cell stage. Dead eggs and embryos were removed regularly. Survival rate was scored as percentage of initial number of fertilized eggs at larval stage 40 [21], i.e. three days after fertilization at 23°C. Morphological malformations and underdevelopment were determined as percentage of at stage 40 survived larvae. Husbandry, breeding and hormone injections were approved by “Landesamt für Natur, Umwelt und Verbraucherschutz Nordrhein-Westfalen (B 952/07).

### RNA extraction

To prepare total RNA ten unfertilized eggs were treated in Precellys24 homogenizer (Peqlab) for 5 sec and RNA extracted with the peqGOLD Total RNA Kit (Peqlab) according to manufacturer's instructions. Extracted RNA was quantified by spectrophotometry on a Nanodrop ND1000 (Peqlab).

### Microarray analysis

The quality of total RNA was assessed on an Agilent chip and by analyzing the cRNA. There was no difference between the RNA samples. Total RNA (100 ng) was converted into double stranded cDNA by using oligo-dT-primers with a T7 RNA polymerase binding site followed by amplification and labeling using the GeneChip 3′ IVT Express Kit (Affymetrix). Labeled cRNA was purified, fragmented and hybridized to *Xenopus tropicalis* Genome Arrays (Affymetrix) followed by washing and staining steps according to the manufacturer's recommendation. Arrays were finally scanned in a GeneChip scanner 3000 G7 (Affymetrix).

Array images were processed to determine signals and detection calls (Present, Absent, Marginal) for each probeset using the Affymetrix GCOS1.4 software (MAS 5.0 statistical algorithm). Arrays were scaled across all probesets to an average intensity of 1000 to compensate for variations in the amount and quality of the cRNA samples and other experimental variables of non-biological origin. Pair-wise comparisons of treated *versus* control samples were carried out with GCOS1.4, which calculates the significance (change p-value) of each change in gene expression based on a Wilcoxon ranking test. To limit the number of false positives, we restricted target identification to probesets with increased or decreased change calls, which exhibited at least one present detection call in the treated/control pair. The data discussed in this publication have been deposited in NCBI's Gene Expression Omnibus under accession number GSE21109.

### Gene Ontology analysis

To identity statistically overrepresented Gene Ontology (GO) terms within a given gene set GO was performed using the Gene Ontology Enrichment Analysis Software Toolkit (GOEAST) [34], which compares the ratio between probesets in the ontology sub-categories and all probesets in given dataset to the ratio between probesets in the ontology sub-categories and all probesets on the microarray platform.

### qRT-PCR

500 ng of mRNA were reverse transcribed using oligo(dT)_20_ (Invitrogen) or random hexamer primer (Applied Biosystems) and the High Capacity cDNA Reverse Transcription Kit from Applied Biosystems according to manufacturer's instructions. qRT-PCR was performed in duplicate using the Power SYBRGreen PCR Master Mix (Applied Biosystems) in an Sequence Detection System 7900HT apparatus (Applied Biosystems). After incubation for 2 min at 50°C and for 10 min at 95°C amplification was performed using the cycle: 95°C for 15 sec; 60°C for 1 min; 40times. The relative amount of mRNA was normalized against ornithine decarboxylase (odc) mRNA as reference. All primers provided by Invitrogen are listed in Table S1.

### Polyadenylation assay

1 µg of mRNA was ligated to 0.4 µg of kinased primer P1 (Eurofins) containing a 3′amino modification to block ligation at this end using T4 RNA Ligase (New England Biolabs) for 30 min at 37°C in a 10 µl volume. The ligated RNA was reverse transcribed using 0.4 µg primer P'1 and the High Capacity cDNA Reverse Transcription Kit (Applied Biosystems) according to manufacturer's instructions. The PCR was performed for 38 cycles and 56°C annealing temperature using gene specific forward and P'1 as reverse primer and the Hot Star Taq Polymerase (Qiagen) in 25 µl according to manufacturer's directions. All primers except P1 were obtained from Invitrogen and are listened in Table S4.

To remove all poly(A) tails, mRNA was treated with RNaseH (New England Biolabs) and 0.3 µg oligo(dT)_20_ for 30 min at 37°C in a 10 µl volume prior to ligation.

### Bioinformatic analysis

For each mRNA 3′UTR sequence was obtained from the UCSC browser via RefSeq transcript ID. 3′UTR length and motif frequency were analyzed using an exact pattern-matching approach implemented in the programming language perl. The algorithm identifies motif occurrence, motif position and total length of each 3′UTR.

To compare our microarray data using the Affymetrix chip with the data using the non-commercial microarray containing 50mer oligonucleotides [15], we searched in Xenbase [46] with the 50mer oligonucleotide sequence the corresponding Affymetrix probeset numbers for the transcripts of the seven categories with an altered adenylation behavior at maturation or upon fertilization.

## Supporting Information

Figure S1 (1.87 MB TIF)

Figure S2 (0.56 MB TIF)

Figure S3 (4.72 MB TIF)

Table S1 (0.01 MB PDF)

Table S2 (0.01 MB PDF)

Table S3 (0.02 MB PDF)

Table S4 (0.01 MB PDF)
