# Supplementary figures and images for: Aging of Xenopus tropicalis Eggs Leads to Deadenylation of a Specific Set of Maternal mRNAs and Loss of Developmental Potential
Source: PLoS One. 2010 Oct 22;5(10):e13532. doi: 10.1371/journal.pone.0013532 (PMC2962626; doi:10.1371/journal.pone.0013532)

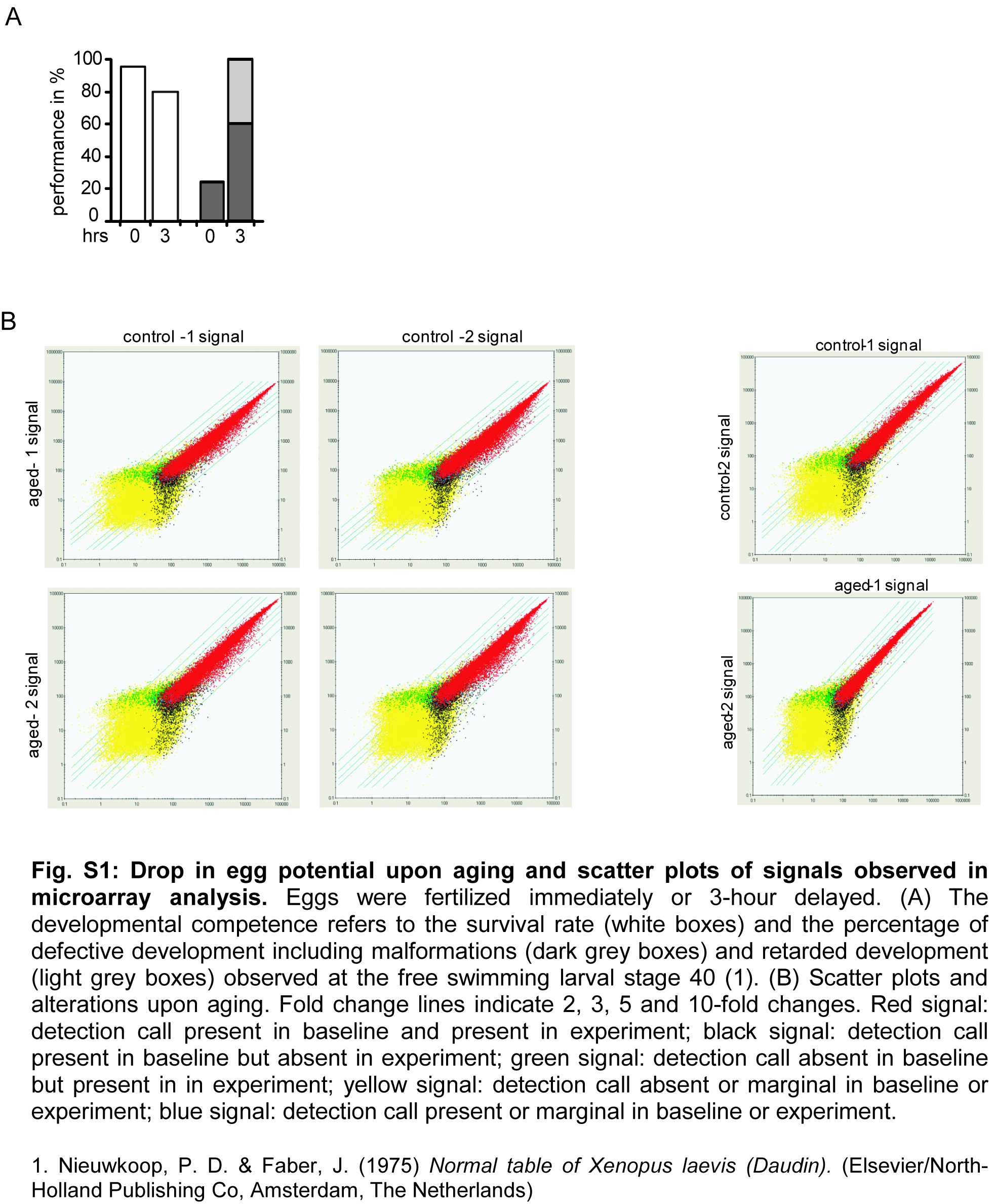

Supplement: Figure S1 — (1.87 MB TIF) [file pone.0013532.s001.tif]

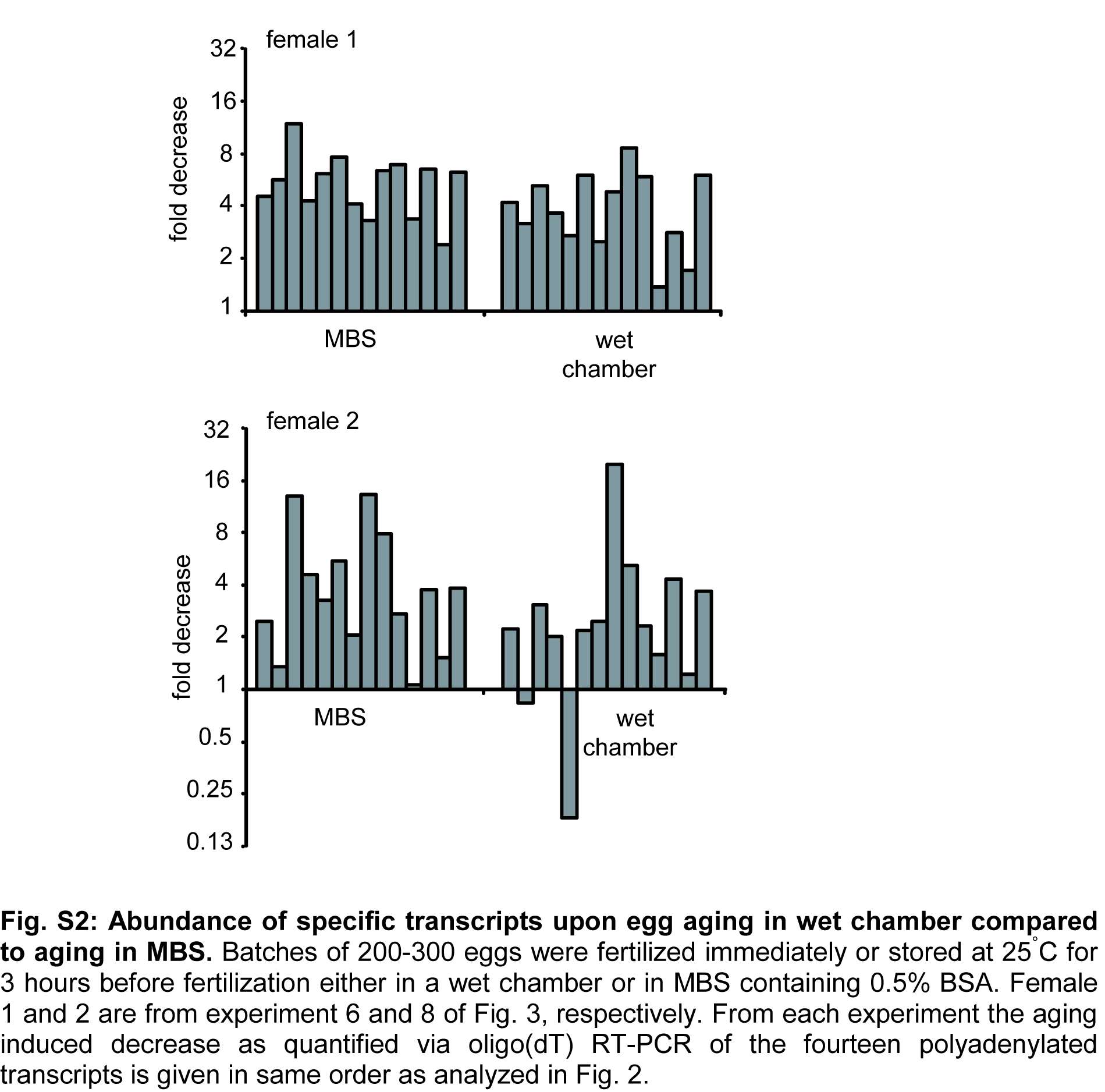

Supplement: Figure S2 — (0.56 MB TIF) [file pone.0013532.s002.tif]

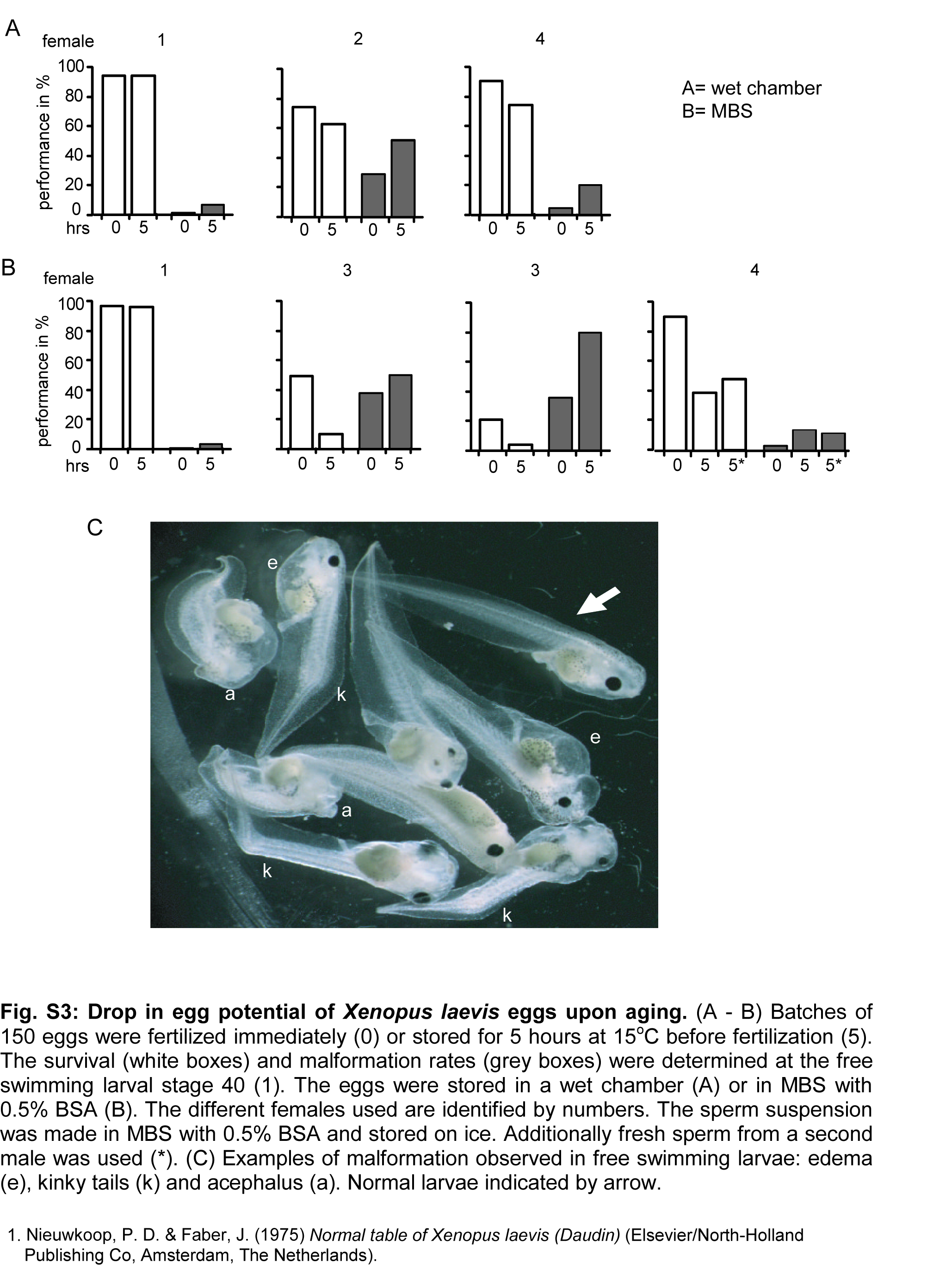

Supplement: Figure S3 — (4.72 MB TIF) [file pone.0013532.s003.tif]
