# Supplementary material for: Aging of Xenopus tropicalis Eggs Leads to Deadenylation of a Specific Set of Maternal mRNAs and Loss of Developmental Potential
Source: PLoS One. 2010 Oct 22;5(10):e13532. doi: 10.1371/journal.pone.0013532 (PMC2962626; doi:10.1371/journal.pone.0013532)
Supplement: Table S1 — (0.01 MB PDF) [file pone.0013532.s004.pdf]

Table S1: Transcripts probed by qRT-PCR.
For each mRNA the sequences of the forward (F) and reverse (R) primers are indicated. Ref-Seq transcript ID and gene title was obtained using the annotation version na30 November 23, 2009 provided by Affymetrix via the corresponding Affymetrix probeset ID. MGC75766 and eno1 are transcripts reported to be deadenylated at maturation as well after fertilization (1) and are thus listed in category 8 (Table S3).

mRNA		Sequences 5´- 3´	Ref-Seq transcript ID	Gene Title	Affymetrix probeset ID
tpi1	F	ATCAACGCCAAGCAATAACC	NM_001114234	triosephosphate isomerase 1	Str.52176.1.S1_at
	R	GGCCGCGTCACTTATTCTAA			
atp5a1	F	TGGAAAGATCTCCGAACAGG	NM_001030439	atp5a1 protein	Str.2749.1.S1_at
	R	AAACTGGTGGCAGAAAAACG			
MGC75766	F	AAAAGGAGAGCATTGCCAAA	NM_203787	ATP synthase subunit B	Str.27670.1.S1_at
	R	GACAGATGGAGCCTGCAGAT			
TGas042k20.1	F	CTTGGTACGTCCCAGTTTGG	NM_203811	hydroxyacyl-Coenzyme A dehydrogenase/3-ketoacyl-Coenzyme A thiolase/enoyl-Coenzyme A hydratase (trifunctional protein), beta subunit	Str.20522.1.S1_s_at
	R	CAAATCGAAACAGACGAGCA			
TGas064h09.1	F	TGTGGCCAAACAAACAAAGA	NM_001016551	prohibitin 2	Str.1047.1.S1_at
	R	GTCAAACAGGGAAGGGAACA			
slc34a2	F	TTGCTGGGAATGATCTGATG	NM_203971	solute carrier family 34 (sodium phosphate), member 2	Str.27160.3.S1_a_at
	R	CGGGGCAGTTTTATTTCAGA			
eif3s9	F	CCTGCTGGATCATCTTGTGA	NM_001016724	eukaryotic translation initiation factor 3, subunit 9 eta, 116kDa	Str.6777.1.S1_at
	R	CGCTGCTTCATTGCTATTCA			
pelo	F	AGTCAAGCTCCGACGATGAT	NM_001004823	pelota homolog	Str.15516.1.S1_a_at
	R	CTATTTCTTTGCCCGGTGAC			
tubb2c	F	GAAGGGGAAGAGGAGGAAAA	NM_001078982	tubulin, beta 2C	Str.8814.1.S1_x_at
	R	TGAAACCGGAGAGAGAAGGA			
sdhb	F	GGCGACGTACAAGGAGAGAG	NM_001126528	succinate dehydrogenase [ubiquinone] iron-sulfur subunit, mitochondrial	StrEns.9993.1.S1_at
	R	CGGAAGTGCGTAGAATCACA			
TNeu126i07.1	F	ATGGGATCACATCAAGCACA	NM_001044430	ubiquinol-cytochrome c reductase complex (7.2 kD) (ucrc)	Str.52223.1.S1_at
	R	CCTTGGATGAAGTGCCAGTC			
mrpl22	F	AGCCGTACGGAATCACAATG	NM_001079446	mitochondrial ribosomal protein L22	Str.17424.1.S1_at
	R	CATGCCACGTCCATGATAAC			
LOC394441	F	CTGTGTCCGGGAATGAAGAT	NM_203519	egg cortical granule lectin	Str.115.1.S1_at
	R	AGGGTCAGCTTCTGGGAAAT			

eno1	F	CTCTCCCAGCATAACCTTGC	NM_203813	enolase 1, (alpha)	Str.5337.1.S1_at
	R	CTACATGCAGGCACCAGTGT			
TEgg053p21.1	F	AACATAAAATCAAGCAACTTTCCA	NM_001004842	MAD2 mitotic arrest deficient-like 2 (yeast)	Str.12948.1.S2_at
	R	GGATAGGCAGAAACTGCTTTC			
nop5	F	GAAGAACCTTCCACCAGCAC	NM_203967	nucleolar protein 5	Str.2215.1.S1_at
	R	TTTCTTTCCTACTCATCATCCTCC			
odc1	F	GACTGTGAGATGGGGTCACA	NM_001005441	ornithine decarboxylase 1	Str.6672.1.S1_at
	R	CACAAGCTACAAATGCTTGAGTG			

1. Graindorge A, Thuret R, Pollet N, Osborne HB, Audic Y (2006) Identification of post-transcriptionally regulated Xenopus tropicalis maternal mRNAs by microarray. Nucleic Acids Res. 34, 986-995.
