# Supplementary material for: Aging of Xenopus tropicalis Eggs Leads to Deadenylation of a Specific Set of Maternal mRNAs and Loss of Developmental Potential
Source: PLoS One. 2010 Oct 22;5(10):e13532. doi: 10.1371/journal.pone.0013532 (PMC2962626; doi:10.1371/journal.pone.0013532)
Supplement: Table S2 — (0.01 MB PDF) [file pone.0013532.s005.pdf]

Table S2: Regulatory elements searched in 3'UTR.
Sequences of the regulatory elements CPE, PAS and ARE used for in silico analysis were obtained from the databases UTR-site (http://www2.ba.itb.cnr.it/UTRSite/)(1) and Transterm (http://uther.otago.ac.nz/)(2) and from motif analysis reported elsewhere (3).

Regulatory element	Motif sequence	Reference
CPE	UUUUUAAU	1
	UUUUUUAU	1
	UUUUAUU	3
	UUUUAAU	3
	UUUUUAU	3
	UUUUAU	2
canonical PAS	AAUAAA	1
non-canonical PAS	AUUAAA	1
	AGUAAA	1
	UAUAAA	1
	CAUAAA	1
	GAUAAA	1
	AAUAUA	1
	AAUACA	1
	AAUAGA	1
	AAUGAA	1
	ACUAAA	1
	AACAAA	1
	UUUAAA	1
ARE	UUAUUUAU	3
	UAUUUAUU	3
	UAUUUAUAA	2
	UAUUUAUAU	2
	UAUUUAUUA	2
	UAUUUAUUU	2

1. Mignone F, et al. (2005) UTRdb and UTRsite: a collection of sequences and regulatory motifs of the untranslated regions of eukaryotic mRNAs. *Nucleic Acids Res.* 33, D141-D146.

2. Jacobs GH, et al. (2009) Transterm: a database to aid the analysis of regulatory sequences in mRNAs. *Nucleic Acids Res.* 37, D72-D76.

3. Graindorge A, Thuret R, Pollet N, Osborne HB and Audic Y (2006). Identification of post-transcriptionally regulated Xenopus tropicalis maternal mRNAs by microarray. *Nucleic Acids Res.* 34, 986-995.
