# Supplementary material for: Aging of Xenopus tropicalis Eggs Leads to Deadenylation of a Specific Set of Maternal mRNAs and Loss of Developmental Potential
Source: PLoS One. 2010 Oct 22;5(10):e13532. doi: 10.1371/journal.pone.0013532 (PMC2962626; doi:10.1371/journal.pone.0013532)
Supplement: Table S3 — (0.02 MB PDF) [file pone.0013532.s006.pdf]

Table S3: Probesets with decreased signal assigned to the categories with differential adenylation behaviour

All probe sets with decreased signal upon aging are listed according to the adenylation behavior categories as defined (1). Category 7 is not included, since there has no sequence been reported in this category. The annotated gene symbol and gene title are given as available in the Affymetrix annotation file na30 November 23, 2009.

(1) Graindorge A, Thuret R, Pollet N, Osborne HB and Audic Y (2006). Identification of post-transcriptionally regulated Xenopus tropicalis maternal mRNAs by microarray. *Nucleic Acids Res.* 34, 986-995.

Category 1 (maturation: depolyadenylated, fertilization: constant)
(71 of 135 decreased)

Probe Set	*Gene Symbol*	*Gene Title*
Str.823.1.S1_at	aco2	aconitase 2, mitochondrial
Str.4269.1.S1_at	aldh9a1	aldehyde dehydrogenase 9 family, member A1
Str.6670.1.S1_at	aldoa	aldolase A, fructose-bisphosphate
Str.648.1.S1_at	arl2	ADP-ribosylation factor-like 2
Str.37821.2.S1_a_at	birc4	birc4 protein
Str.6989.1.S1_at	c3orf37	chromosome 3 open reading frame 37
Str.1698.1.S1_at	ccdc103	coiled-coil domain containing 103
Str.10998.1.S1_at	cndp2	CNDP dipeptidase 2 (metallopeptidase M20 family)
Str.4820.1.S1_at	eef1a1o	eukaryotic translation elongation factor 1 alpha 1, oocyte form
Str.47229.1.A1_x_at	eef1a1o	eukaryotic translation elongation factor 1 alpha 1, oocyte form
Str.8415.1.S1_at	eif3l	eukaryotic translation initiation factor 3, subunit L
Str.27072.2.S1_at	epc1	Enhancer of polycomb homolog 1
Str.27072.1.S1_at	epc1	enhancer of polycomb homolog 1
Str.5017.1.S1_at	exosc2	exosome component 2
Str.6679.1.S1_at	g3bp2	GTPase activating protein (SH3 domain) binding protein 2
Str.1620.1.S1_at	gamt	guanidinoacetate N-methyltransferase
Str.8069.1.S1_at	gapdh	glyceraldehyde-3-phosphate dehydrogenase
AFFX-Str-gapdh-M_at	gapdh	glyceraldehyde-3-phosphate dehydrogenase
AFFX-Str-gapdh-5_at	gapdh	glyceraldehyde-3-phosphate dehydrogenase
AFFX-Str-gapdh-3_at	gapdh	glyceraldehyde-3-phosphate dehydrogenase
Str.8025.1.S1_a_at	krt18	keratin 18
Str.8680.1.S1_at	ldhb	lactate dehydrogenase B
Str.1431.1.S1_at	LOC100124857	hypothetical protein LOC100124857
Str.22053.1.S1_at	LOC100125021	hypothetical protein LOC100125021
Str.2314.1.S1_at	LOC100125169	Hypothetical protein LOC100125169
Str.18325.1.A1_at	LOC100127637	hypothetical protein LOC100127637
Str.15051.1.S1_at	LOC100135217	Hypothetical protein LOC100135217
Str.1340.2.S1_at	LOC100145083	hypothetical protein LOC100145083
Str.21798.1.S1_at	LOC100216164	hypothetical protein LOC100216164
Str.11945.1.S1_at	LOC496692	hypothetical LOC496692
Str.22124.1.S1_at	LOC496785	hypothetical LOC496785
Str.17221.1.S1_at	LOC549152	hypothetical protein LOC549152
Str.21546.1.S1_at	LOC549289	hypothetical protein LOC549289
Str.3137.1.S1_at	LOC549492	hypothetical protein LOC549492
Str.84.1.S1_at	mdh1	malate dehydrogenase 1, NAD (soluble)
Str.6413.1.S1_a_at	MGC69505	MGC69505 protein
Str.287.1.S1_at	MGC75803	hypothetical protein MGC75803
Str.710.1.S1_at	MGC75949	hypothetical protein MGC75949

Str.830.1.S1_at	MGC75970	hypothetical protein MGC75970
Str.6893.1.S1_at	MGC76063	COP9 signalosome subunit 5
Str.20889.1.S1_s_at	MGC76063	COP9 signalosome subunit 5
Str.6796.1.S1_at	MGC76097	carnitine/acylcarnitine translocase
Str.24372.1.S1_s_at	MGC79787	MGC79787 protein
Str.52165.1.S1_s_at	MGC89973	MGC89973 protein
Str.1295.1.S1_at	MGC89973	MGC89973 protein
Str.1362.1.S1_a_at	MGC97811	MGC97811 protein
Str.11979.1.S1_at	mrpl23	mitochondrial ribosomal protein L23
Str.10186.1.S1_at	ndufv2	NADH dehydrogenase (ubiquinone) flavoprotein 2, 24kDa
Str.1896.1.S2_at	nola2	nucleolar protein family A, member 2
Str.11112.1.S1_at	oprs1	oprs1 protein
Str.6228.1.S1_at	pcna	pcna protein
Str.7593.1.S1_at	psma7	proteasome (prosome, macropain) subunit, alpha type 7
Str.13591.1.S1_at	psmb1	proteasome (prosome, macropain) subunit, beta type, 1
Str.1435.1.S1_at	psmd13	proteasome (prosome, macropain) 26S subunit, non-ATPase, 13
Str.17153.1.A1_a_at	rbbp7	retinoblastoma binding protein 7
Str.51721.1.S1_at	rbpms2	RNA binding protein with multiple splicing 2
Str.6969.1.S1_at	rps6	ribosomal protein S6
Str.2049.3.S1_a_at	slc25a3	solute carrier family 25 (mitochondrial carrier; phosphate carrier), member 3
Str.2049.2.S1_a_at	slc25a3	solute carrier family 25 (mitochondrial carrier; phosphate carrier), member 3
Str.27202.1.S1_at	smarca5	SWI/SNF related, matrix associated, actin dependent regulator of chromatin, subfamily a, member 5
Str.9957.1.S1_at	spop	speckle-type POZ protein
Str.9533.2.S1_a_at	sppl3	signal peptide peptidase 3
Str.290.1.S1_s_at	TEgg033f08.1	MGC69396 protein
Str.10791.1.S1_at	TEgg046m03.1	F-box protein 5
Str.10339.1.S1_s_at	TGas057c05.1	replication protein A1, 70kDa
Str.1047.1.S1_at	TGas064h09.1	prohibitin 2
Str.1679.1.S1_a_at	TNeu089n18.1	ribosomal protein S23
Str.8172.1.S1_at	TNeu104f20.1 /// wfikkn1	UPF0585 protein C16orf13 homolog /// WAP, follistatin/kazal, immunoglobulin, kunitz and netrin domain containing 1
Str.7616.1.S1_at	trnt1	tRNA nucleotidyl transferase, CCA-adding, 1
Str.3011.1.S1_at	tsta3	tissue specific transplantation antigen P35B
Str.3286.1.S1_at	TTpA010e06.1	peroxiredoxin 2

Category 2 (maturation: polyadenylated, fertilization: constant)
(9 of 54 decreased)

Probe Set	*Gene Symbol*	*Gene Title*
Str.9724.1.S1_at	aifm1	apoptosis-inducing factor, mitochondrion-associated, 1
Str.5809.1.S1_at	capn1	calpain 1, (mu/I) large subunit
Str.14315.1.A1_a_at	LOC100144949	hypothetical protein LOC100144949
Str.5645.1.S1_at	MGC107795	MGC107795 protein
Str.50662.1.A1_s_at	MGC107795	MGC107795 protein
Str.50662.1.A1_x_at	MGC107795	MGC107795 protein
Str.27234.1.S2_a_at	MGC76214	hypothetical protein MGC76214
Str.286.1.S2_at	TGas142e24.1	high-mobility group 20A
Str.5927.1.S2_at	ube2h	ubiquitin-conjugating enzyme E2H (UBC8 homolog)

Category 3 (maturation: constant, fertilization: polyadenylated)
(6 of 18 decreased)

Probe Set	*Gene Symbol*	*Gene Title*
Str.10827.1.S2_at	capza2	capping protein (actin filament) muscle Z-line, alpha 2
Str.10827.1.S1_at	capza2	capping protein (actin filament) muscle Z-line, alpha 2

Str.5921.1.S1_at	e2f3	E2F transcription factor 3
Str.10770.1.S1_at	LOC779588	hypothetical protein LOC779588
Str.299.1.S1_at	timm13	translocase of inner mitochondrial membrane 13 homolog (yeast)
Str.11786.1.A1_at	znf574	zinc finger protein 574

Category 4 (maturation: polyadenylated, fertilization: depolyadenylated)
(3 of 22 decreased)

Probe Set	*Gene Symbol*	*Gene Title*
Str.8413.1.S1_at	atic	5-aminoimidazole-4-carboxamide ribonucleotide formyltransferase/IMP cyclohydrolase
Str.15861.1.S1_at	rabep1	rabaptin, RAB GTPase binding effector protein 1
Str.1679.1.S1_a_at	TNeu089n18.1	ribosomal protein S23

Category 5 (maturation: constant, fertilization: depolyadenylated)
(14 of 33 decreased)

Probe Set	*Gene Symbol*	*Gene Title*
Str.27623.1.S1_at	adrm1	adhesion regulating molecule 1
Str.1620.1.S1_at	gamt	guanidinoacetate N-methyltransferase
Str.15297.1.S1_at	grhpr	grhpr protein
Str.37852.3.A1_a_at	LOC100145798	hypothetical protein LOC100145798
Str.5678.1.S1_a_at	LOC447981	hypothetical protein LOC447981
Str.1999.1.S1_at	LOC549006	hypothetical protein LOC549006
Str.20792.1.S1_at	LOC733864	hypothetical protein LOC733864
Str.6067.1.S1_at	MGC75699	ribosomal protein S3
Str.989.1.S1_at	MGC76130	hypothetical protein MGC76130
Str.1295.1.S1_at	MGC89973	MGC89973 protein
Str.52165.1.S1_s_at	MGC89973	MGC89973 protein
Str.15833.1.S1_at	prdx3	prdx3 protein
Str.24756.1.S1_at	TGas028n11.1	formiminotransferase cyclodeaminase
Str.6150.1.A1_at	vdac2	voltage-dependent anion channel 2

Category 6 (maturation: depolyadenylated, fertilization: polyadenylated)
(28 of 50 decreased)

Probe Set	*Gene Symbol*	*Gene Title*
Str.11896.2.S1_at	---	---
Str.10460.1.S1_at	agpat4	1-acylglycerol-3-phosphate O-acyltransferase 4 (lysophosphatidic acid acyltransferase, delta)
Str.10583.2.S1_at	brca1	breast cancer 1, early onset
Str.1811.1.S1_at	cdk9	cyclin-dependent kinase 9
Str.6567.1.S1_at	cfl1	cofilin 1 (non-muscle)
Str.6885.1.S1_at	cox5a	cytochrome c oxidase, subunit Va
Str.22110.1.S1_at	LOC100170577	hypothetical protein LOC100170577
Str.24802.1.S1_at	LOC548854	hypothetical protein LOC548854
Str.15407.1.S1_at	lsm1	LSM1 homolog, U6 small nuclear RNA associated (S. cerevisiae)
Str.16611.1.S1_at	MGC107838	MGC107838 protein
Str.6205.1.S1_at	MGC75715	hypothetical protein MGC75715
Str.6205.1.S2_at	MGC75715	hypothetical protein MGC75715
Str.4770.1.S1_at	MGC75981	Hypothetical protein MGC75981
Str.1276.1.S1_at	MGC76019	hypothetical protein MGC76019
Str.27671.1.S1_a_at	MGC76078	N-acetyltransferase 5
Str.10709.1.S1_at	MGC89693	MGC89693 protein
Str.24766.1.S1_at	pdlim1	PDZ and LIM domain 1 (elfin)
Str.6229.1.A1_at	pou5f1.1	POU class 5 homeobox 1, gene 1
Str.6646.1.S1_at	rnf121	ring finger protein 121

Str.1730.1.S1_at	ruvbl2	RuvB-like 2
Str.1730.1.S1_a_at	ruvbl2	RuvB-like 2
Str.524.1.S2_at	snip1	Smad nuclear interacting protein 1
Str.492.1.S1_a_at	surf4	surfeit gene 4
Str.6655.1.S1_at	TEgg054b02.1	mitochondrial outer membrane protein TOM40
Str.286.1.S2_at	TGas142e24.1	high-mobility group 20A
Str.17179.1.S1_at	tk1	thymidine kinase 1, soluble
Str.3387.1.S2_at	tmpo	thymopoietin
Str.17226.1.S1_at	xylt2	xylosyltransferase II

Category 8 (maturation: depolyadenylated, fertilization: depolyadenylated)
(15 of 17 decreased)

Probe Set	*Gene Symbol*	*Gene Title*
Str.5646.1.S1_at	arbp	acidic ribosomal phosphoprotein P0
Str.24243.1.S1_at	cks1b	CDC28 protein kinase regulatory subunit 1B
Str.5337.1.S1_at	eno1	enolase 1, (alpha)
Str.9960.1.S1_at	fkbp6	FK506 binding protein 6
Str.1869.1.S1_at	gyg1	glycogenin 1
Str.1374.1.S1_s_at	LOC549795	hypothetical protein LOC549795
Str.1374.1.S1_x_at	LOC549795	hypothetical protein LOC549795
Str.27670.1.S1_at	MGC75766	ATP synthase subunit B
Str.1295.1.S1_at	MGC89973	MGC89973 protein
Str.52165.1.S1_s_at	MGC89973	MGC89973 protein
Str.1295.1.S1_at	MGC89973	MGC89973 protein
Str.9100.1.S1_a_at	TGas044l18.1	60S ribosomal protein L35
Str.9100.3.S1_a_at	TGas044l18.1	60S ribosomal protein L35
Str.1679.1.S1_a_at	TNeu089n18.1	ribosomal protein S23
Str.6703.1.S1_at	TNeu119p20.1	ribosomal protein SA
