# Supplementary material for: Aging of Xenopus tropicalis Eggs Leads to Deadenylation of a Specific Set of Maternal mRNAs and Loss of Developmental Potential
Source: PLoS One. 2010 Oct 22;5(10):e13532. doi: 10.1371/journal.pone.0013532 (PMC2962626; doi:10.1371/journal.pone.0013532)
Supplement: Table S4 — (0.01 MB PDF) [file pone.0013532.s007.pdf]

Table S4: Oligos used for RL-PAT

The ligated primer P1 is modified by a 5´phosphate and 3´amino group. The complementary P´1 primer was used for reverse transcription and as reverse primer in PCR. Gene specific forward primers are indicated for each analyzed transcript. Ref-Seq transcript ID was obtained using the annotation version na30 November 23, 2009 provided by Affymetrix via the corresponding Affymetrix probeset ID.

mRNA	Sequences 5´- 3´	Ref-Seq transcript ID
P1	P-GGTCACCTTGATCTGAAGC-NH2	
P´1	GCTTCACATCAAGGTGACCTTTTT	
atp5a1	TGGAAAGATCTCCGAACAGG	NM_001030439
tpi1	ATCAACGCCAAGCAATAACC	NM_203787
eif3s9	CCTGCTGGATCATCTTGTGA	NM_001016724
grhpr	AAGCAGCATGACATCTAATGGA	NM_001007895
odc1	GACTGTGAGATGGGGTCACA	NM_001005441
melk	TCCTCTCTGATTCACATGGTGT	NM_001016390
TEgg053p21.1	AACATAAAATCAAGCAACTTTCCA	NM_001004842
nop5	AGACTCACAATGACTCTTTAGCATAAACC	NM_203967
tmem85	TTGGCATTAGCTGTCTACAAGTG	NM_203545
